# Higher efficacy and reduced adverse reactions in neoadjuvant chemotherapy for breast cancer by using pegylated liposomal doxorubicin compared with pirarubicin

**DOI:** 10.1038/s41598-020-80415-w

**Published:** 2021-01-08

**Authors:** Weifang Liu, Wei Chen, Xiuxiang Zhang, Peng Zhao, Zhimin Fan, Lirong Bi, Di Wu, Sijie Li, Ming Yang, Tong Fu, Dong Song, Bing Han, Gang Zhao, Ye Du, Aiping Shi

**Affiliations:** 1 Department of Breast Surgery, First Hospital of Jilin University, Changchun, China; 2 Department of Breast Surgery, Song Yuan Central Hospital, Changchun, China; 3 Department of Thyroid and Breast Surgery, Affiliated Hospital of Jining Medical University, Shandong, China; 4 Department of Pathology, First Hospital of Jilin University, Changchun, China

**Keywords:** Breast cancer, Toxicology

## Abstract

The present study aimed to investigate the efficacy and toxicity of pegylated liposomal doxorubicin (PLD) in preoperative neoadjuvant chemotherapy for patients with breast cancer by comparing with conventional anthracycline. This study is a non-randomized controlled trial. Prospective analysis was conducted after matching as required. A total of 146 patients with confirmed diagnosis of breast cancer by histopathological examinations were enrolled into the observation group and control group in 1:1 ratio. Each of the cases in the observation group was required to correspond to another in the control group according to the requirements including age, molecular subtype, axillary node status, and regimen of the preoperative neoadjuvant chemotherapy. The chemotherapy was based on regimens consisting of anthracyclines, paclitaxel or docetaxel, and/or platinum. PLD was used at least twice in the observation group, with traditional anthracycline as a contrast in the control group. Clinical responses as well as cardiac side effects and other adverse reactions were evaluated by clinical and imaging examinations such as electrocardiogram (ECG) and color Doppler ultrasound during the chemotherapy. Pathologic examinations were performed following the surgeries after preoperative neoadjuvant chemotherapy. All the patients in both groups completed the preoperative neoadjuvant chemotherapy according to their original regimens. The postoperative pathological evaluation revealed a higher pathologic complete response (PCR) rate and significantly more patients of grade V of the Miller-Payne grading system in the observation group as compared to the control group (*p* = 0.047). In addition, the observation group recorded an evidently lower occurrence of the adverse cardiac events (*p* = 0.014), ECG changes (*p* = 0.048), and the relatively severe adverse reactions such as myelosuppression. Compared with conventional anthracycline drugs, PLD has a better pathologic response and safety performance, as well as a similar clinical effectiveness in preoperative neoadjuvant chemotherapy for breast cancer.

## Introduction

Breast cancer is one of the most common malignant tumors in women worldwide, with a mortality rate only lower than that of lung cancer among all the malignancies. Every year, more than 1.3 million women in the world are newly diagnosed with breast cancer, and about 500,000 cases die of this life-threatening disease^1^. Breast cancer management towards a favorable prognosis requires comprehensive measures, among which chemotherapy has always been used as a key part due to its substantial clinical effects in disease control.

Neoadjuvant therapy is typically used for the management of local malignant tumors prior to surgery and other treatment^2^. There are three types of neoadjuvant therapy for breast cancer—preoperative neoadjuvant chemotherapy, targeted therapy and endocrine therapy. A number of clinical trials showed that 80% of the patients with breast cancer achieved a significant reduction in tumor size, and about 10–20% obtained pathologic complete response (PCR) by preoperative neoadjuvant chemotherapy^3^. National Comprehensive Cancer Network (NCCN) guidelines also recommend preoperative neoadjuvant chemotherapy as a routine for stage II and part of stage III breast cancer^4^.

However, a variety of side effects, even toxic responses concomitant with chemotherapy have put a limit to the clinical application of the antineoplastics, resulting in the restricted use of some potent chemotherapeutic drugs despite of their promising future in improving the survival rate for patients with breast cancer. Toxic reactions caused by chemotherapy varies with the agents, but most commonly nausea, vomiting, myelosuppression, and even some severer side effects that may discontinue the treatment. Cardiotoxicity of a drug is usually confirmed when congestive heart failure occurs accompanied by relevant clinical symptoms after treatment, or left ventricular ejection fraction (LVEF) is less than 55% or decreases by more than 10% compared with the reference value although absent of clinical symptoms^5,6^. The clinical symptoms suggesting congestive heart failure include but not limited to lung moist rale, pretibial edema of both legs, and cyanosis of lip or mouth.

There are many kinds of antineoplastics used for chemotherapy. Anthracyclines, taxanes and targeted drugs are frequently employed as adjuvant drugs in breast cancer treatment. Anthracyclines, for instance, has been constantly playing an irreplaceable role in chemotherapy, particularly for the patients with locally advanced breast cancer^7^. A large number of clinical studies have also confirmed that anthracyclines are currently a routine by combining with taxus in preoperative neoadjuvant chemotherapy^8^. However, the cardiotoxicity caused by anthracycline agents is still considered by practitioners as a great challenge to their clinical application, particularly for elderly patients and those receiving anthracycline-based multi-course chemotherapy owing to the potential risk of heart failure^9,10^. Fortunately, the progress in pharmaceutical technology over the recent years has contributed to the development of a large number of novel agents to tackle these problems. Pegylated liposomal doxorubicin (PLD), a new dosage form of doxorubicin which is encapsulated by liposome, can form a stable three-dimensional structure (stealth liposome) by pegylation on the surface of liposome. In the treatment of metastatic breast cancer, PLD showed a similar efficacy as doxorubicin, but a lower incidence of nausea, vomiting, and cardiotoxicity^11^. Therefore, in the present study, we aimed to evaluate the efficacy, cardiotoxicity and other side effects of PLD in preoperative neoadjuvant chemotherapy for breast cancer by comparing with traditional anthracycline agents. ClinicalTrials.gov ID: NCT02953184 (01/11/2016) and Unique Protocol ID: 2016yx238.

## Materials and methods

### Patients

From September 2016 to December 2019, a total of 146 patients with breast cancer were recruited in the Department of Breast Surgery, the First Hospital of Jilin University. The admission criteria were as follows: (1) female patients of 18 years old or above, with an initial diagnosis of stage I–III breast cancer confirmed by histopathological examinations; (2) tolerable for preoperative neoadjuvant chemotherapy; (3) Eastern Cooperative Oncology Group (ECOG) physical score 0–1, LVEF ≥ 55%, and no abnormality observed in ECG. (4) Other necessary blood tests were also carried out with the results meeting the requirements for chemotherapy—white blood cells ranging from 3.5 × 10^9^ to 9.5 × 10^9^; aspartate aminotransferase 13.0–35.0 (U/L); and alanine aminotransferase 7.0–40.0 (U/L).

The exclusion criteria were: (1) patients with severe systemic infection or diseases; (2) allergic or intolerant to chemotherapy drugs or their excipients; (3) patients of child-bearing age who refused to take appropriate contraceptive measures during the treatment and one year thereafter; (4) patients with conditions not suitable for this study according to the judgment of the researchers, such as potential mental illness, poor compliance, or residential address far away from the research center.

In NSABP B27, 2411 patients were enrolled, of which the PCR rate of AC-T protocol (anthracyclines combined with cyclophosphamide followed by sequential Taxus) was 26.1%^12^. In a preoperative neoadjuvant chemotherapy phase II clinical study, 35 patients were enrolled, with the result showing the PCR rate of 17% in the scheme of pegylated liposome adriamycin plus cyclophosphamide followed by sequential Taxus. In this study, β error was 0.2, and α error was 0.05, with a boundary value (δ) of 0.1. According to the data of rescue treatment, assuming the overall effective rate of treatment was obtained at a power of 80% and a variance of 2, and the minimum sample size was calculated according to 1:1 matching, therefore the number of enrolled patients was set as 80 for each of the groups, of which 10% of the patients were allowed to fall off during the treatment.

### Treatment plan

The chemotherapy schemes for both groups were based on a similar combination use of the agents such as anthracyclines, paclitaxel or docetaxel, platinum and molecular targeted drugs, specifically, AC-T protocol, anthracyclines combined with cyclophosphamide followed by sequential Taxus and trastuzumab (AC-TH), anthracyclines combined with Taxus and cyclophosphamide (TAC), as well as anthracyclines combined with Taxus (TA). 68.5% (50 cases), 17.8% (13 cases), 5.5% (4 cases) and 8.2% (6 cases) of the patients in the observation group received AC-T, AC-TH, TAC/TA or platinum, respectively, corresponding to 74% (54 cases), 15.1% (11 cases), 2.7% (2 cases) and 8.2% (6 cases) in the control group. In addition, PLD was intravenously administrated to the patients in the observation group, 35 mg/m^2^, at least twice for each one, while pirarubicin, a representative agent of traditional anthracyclines, was infused intravenously at 60 mg/m^2^ as the control for those in the control group. Dextrine and other necessary measures were allowed in this study for heart protection and prevention from potential myocardial injury. Symptomatic treatment such as antidiarrheals, antiemetics, analgesics, granulocyte stimulating factors and other supportive treatment could also be used if necessary. For cases with evidence suggesting cardiotoxicity attributed to PLD or pirarubicin for more than 3 weeks after the start of the next cycle, the subjects should quit the study if the examinations didn’t show a full recovery from the cardiotoxicity. For the patients failed to complete the established chemotherapy regimens, a protocol without anthracyclines should be used to replace the original one till the completion of the treatment, or the treatment should be discontinued based on the judgement of the oncologists. The patients in both of the observation group and control group underwent surgical treatment after preoperative neoadjuvant chemotherapy, including modified radical mastectomy [51 cases (69.9%) vs. 57 cases (78.1%)], breast conserving surgery [11 cases (15.1%) vs. 6 cases (8.2%)], breast cancer simple resection plus sentinel lymph node biopsy [7 cases (9.6%) vs. 10 cases (13.7%)], and breast cancer prosthesis implantation [4 cases (5.5%) vs. 0 cases (0%)]. All the patients achieved R0 resection.

### Efficacy and pathological assessment

The tumor was evaluated every 3 weeks until the disease progressed. The clinical evaluation was performed after 6–8 rounds of preoperative neoadjuvant chemotherapy but before surgeries. Besides, the objective remission rate of tumor was evaluated by color Doppler ultrasound before each cycle of chemotherapy. The clinical effect of the chemotherapy is assessed in accordance with the Response Evaluation Criteria In Solid Tumors (RECIST)^13^: *Complete response (CR)* the breast and armpit on the affected side are not touched with obvious masses and enlarged lymph nodes; *Partial response (PR)* the sum of the largest diameters of the targeted lesions decreases by ≥ 30% as compared to the baseline sum diameters; *Stable disease (SD)* the sum of the maximum diameters of the targeted lesions decreases by < 30% or increases by < 20% ; *Progressive disease (PD)* the sum of the largest diameters of the targeted lesions increases by more than 20%, or new lesions appear, or enlarged lymph nodes are touched in the armpit where no enlargement of lymph nodes was detected before chemotherapy. In addition, PCR rate and Miller–Payne grading was employed for pathological evaluation following the surgeries. Pathological response was defined as PCR in the patients with complete clinical responses and no residual invasive disease in pathological evaluation of the surgical breast specimen. Specimens with only residual ductal carcinoma in situ (DCIS) were classified as PCR (ypT0/is)^14^. In the present study, patients with a Miller–Payne grade of V were classified as major pathologic response, patients with a grade of III or IV were considered as non-major pathologic response, and those with a grade of I or II as poor pathologic response.

### Toxicity and adverse reactions

The safety of the chemotherapeutic agents was monitored throughout the study based on the clinical manifestations of the patients. Hematological and biochemical analyses were carried out in a locally certified laboratory. Echocardiography and electrocardiograph (ECG) were performed before admission and after the first, second, third, fourth, sixth and eighth cycles of chemotherapy, respectively. Adverse events as well as their severity, frequency, impact on the investigational treatment, and outcomes were all recorded. According to the World Health Organization (WHO) classification standard for adverse reactions, the adverse reactions were classified as five grades—0, I, II, III and IV, respectively, demonstrating a positive correlation between the toxicity and the grading. In the follow-ups, the toxicity and other side effects were also traced by observation of the clinical manifestations during chemotherapy, as well as outpatient examinations and telephone consultant after discharge from the hospital.

### Statistical analysis

Spss25.0 was employed for statistical analysis, with Chi square test used for counting data. When the total number of cases (n) is ≥ 40 with all the theoretical frequencies (T) ≥ 5, Pearson chi square shall be used for analysis; continuity correction shall be conducted for n ≥ 40 with one T ≥ 1 and < 5, and the results produced by continuity correction shall prevail; Fisher’s exact test shall be used for n ≥ 40 along with two or more T ≥ 1 and < 5, n < 40, or T < 1. *p* < 0.05 was considered statistically significant in this study.

### Ethics approval

The project was approved by the Ethics Committee of Jilin University with an approval number 2016-341. All the procedures performed in this study were in accordance with the Declaration of Helsinki.

### Informed consent

Thoroughly understanding the process of this study, all the patients signed the written informed consent.

## Results

In this study, seven patients dropped out because they failed to undergo the surgery in our hospital. The total number of the subjects who eventually completed the study was 146, 73 cases for each group. The two groups were consistent with, and comparable to each other in the age of the patients, menstrual status, as well as tumor T staging, axillary lymph node status, clinical staging, and molecular subtypes according to the pathological diagnosis, as shown in Table 1.

**Table 1:** Comparison of the characteristics of the patients between observation group and control group.

	Observation group (n = 73)	Control group (n = 73)	Test value	*p*-value
Age (year), average and range	49.3 (29–72)	49.8 (31–66)	z = − 0.763	0.446
**ECOG score**			χ^2^ = 0.00	1.00
0	68	68		
1	5	5		
**Menstrual status**			χ^2^ = 0.00	1.00
Premenopausal	40	40		
Postmenopausal	33	33		
**Tumor T staging**			χ^2^ = 0.948	0.861
T1	18	15		
T2	50	51		
T3	4	6		
T4	1	1		
**Axillary lymph node status**			χ^2^ = 1.829	0.490
N0	14	12		
N1	58	57		
N2	1	4		
**Clinical staging**			χ^2^ = 2.903	0.259
I	2	2		
II	67	61		
III	4	10		
**ER status**			χ^2^ = 1.118	0.732
Negative	26	28		
Positive	47	45		
**HER-2 status**			χ^2^ = 0.590	0.442
Negative	53	57		
Positive	20	16		
**Molecular subtypes**			χ^2^ = 1.391	0.747
Luminal A	5	2		
Luminal B	42	43		
HER-2	11	11		
Triple negative	15	17		
**Chemotherapy regimen**			χ^2^ = 1.035	0.825
AC-T	50	54		
AC-TH	13	11		
TAC/TA	4	2		
Containing platinum	6	6		

*ECOG score* Eastern Cooperative Oncology Group (ECOG) physical score, *ER* estrogen receptor, *HER-2* human epidermal growth factor receptor, *AC-T* anthracyclines combined with cyclophosphamide followed by sequential Taxus, *AC-TH* anthracyclines combined with cyclophosphamide followed by sequential Taxus and trastuzumab, *TAC/TA* anthracyclines combined with Taxus and cyclophosphamide/anthracyclines combined with Taxus.

### Clinical and pathologic response

**Figure 1:**
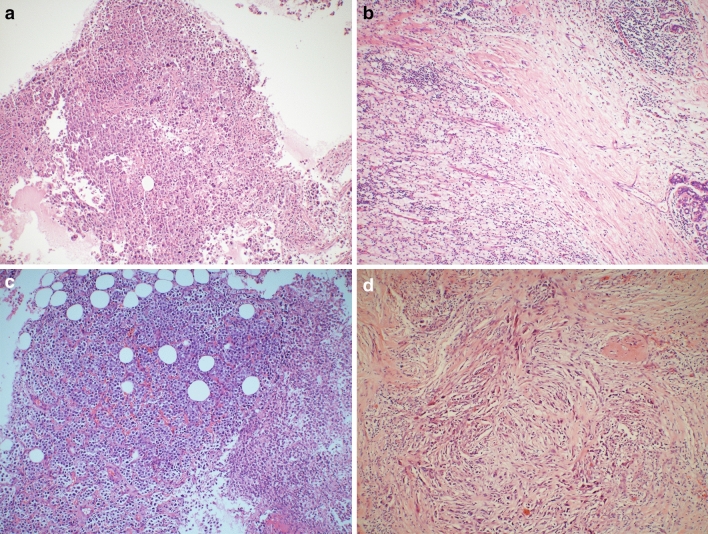
Pathological pictures (hematoxylin–eosin stain, original magnification × 100). (**a**) The preoperative puncture pathology of a patient in the observation group, showing a large number of tumor cells, (**b**) postoperative pathology, showing a large number of inflammatory cells, without tumor cells; (**c**) the corresponding preoperative puncture pathology picture of a patient in the control group, showing a large number of tumor cells, (**d**) the postoperative pathology, showing the residual tumor cells.

Clinical response following the preoperative neoadjuvant chemotherapy and postoperative pathology were evaluated for both groups. The results showed no significant difference in clinical efficacy between the two groups, as shown in Table 2 and Fig. 1. In contrast, the postoperative pathology demonstrated a higher PCR rate (Table 3), as well as a remarkably higher rate of major pathologic response as per Miller-payne grading (Table 4), in the observation group as compared to the control group.

**Table 2:** Clinical response following the preoperative neoadjuvant chemotherapy (unit: case).

	Evaluation of clinical response				Total	ORR (CR + PR)	Chi-square	*p*-value
	CR	PR	SD	PD				
Observation group	3	61	7	2	73	64		
Control group	2	63	8	0	73	65	0.014	0.906
Total	5	124	15	2	146	129		

*CP* complete response, *PR* partial response, *SD* stable disease, *PD* progressive disease, *ORR* objective response rate.

**Table 3:** Postoperative pathological evaluation (unit: case).

	Pathological evaluation		Total	Chi-square	*p*-value
	PCR	non-PCR			
Observation group	12 (16.4%)	61 (83.6%)	73	2.281	
Control group	6 (8.2%)	67 (91.8%)	73		0.131
Total	18	128	146		

*PCR* pathological complete response, *non-PCR* not pathological complete response.

**Table 4:** Miller–Payne grading of postoperative pathology (unit: case).

	Miller–Payne grading				
	Observation group (%)	Control group (%)	Total	Chi-square	*p*-value
Poor pathologic response (I/II)	17 (23.3)	25 (34.3)	42		
Non-major pathologic response (III/IV)	40 (54.8)	42 (57.5)	82	6.118	0.047
Major pathologic response (V)	16 (21.9)	6 (8.2)	22		
Total	73	73	146		

### Adverse cardiac reactions

Adverse cardiac reactions, primarily tachycardia, dyspnea and suffocation, were recorded in both groups when the patients conducted light physical work, but with a significantly lower occurrence in the observation group compared with the control group. Similarly, ECG changes, mainly ST segment changes, flat or inversed T-wave, were observed in more patients in the control group (Table 5). The symptoms and ECG changes were recovered in the patients of both groups after resting and avoiding strenuous physical exertion. Echocardiography suggested that the LVEF level was not less than 55% or decreased by more than 10% in either of the two groups compared with the baseline data.

**Table 5:** Adverse reactions with a high incidence following the preoperative neoadjuvant chemotherapy (unit: case).

Adverse reactions		Observation group			Control group			Chi-square	*p*-value
		II	III	IV	II	III	IV		
Nausea and vomiting		5	3	0	16	9	0	11.361	0.001
Alopecia		16	5	0	24	20	0	14.835	< 0.001
Oral ulcer		17	6	0	4	2	0	12.479	< 0.001
Fatigue		12	1	0	19	1	0	1.925	0.165
Myelosuppression		11	2	0	22	8	0	9.582	0.002
Rash		20	3	0	4	1	0	22.178	< 0.001
HFS		20	9	0	7	0	0	17.926	< 0.001
**Cardiotoxicity**									
Symptoms		3			12			6.018	0.014
ECG changes		8			17			3.909	0.048

*HFS* hand-foot syndrome.

### Other adverse reactions

**Figure 2:**
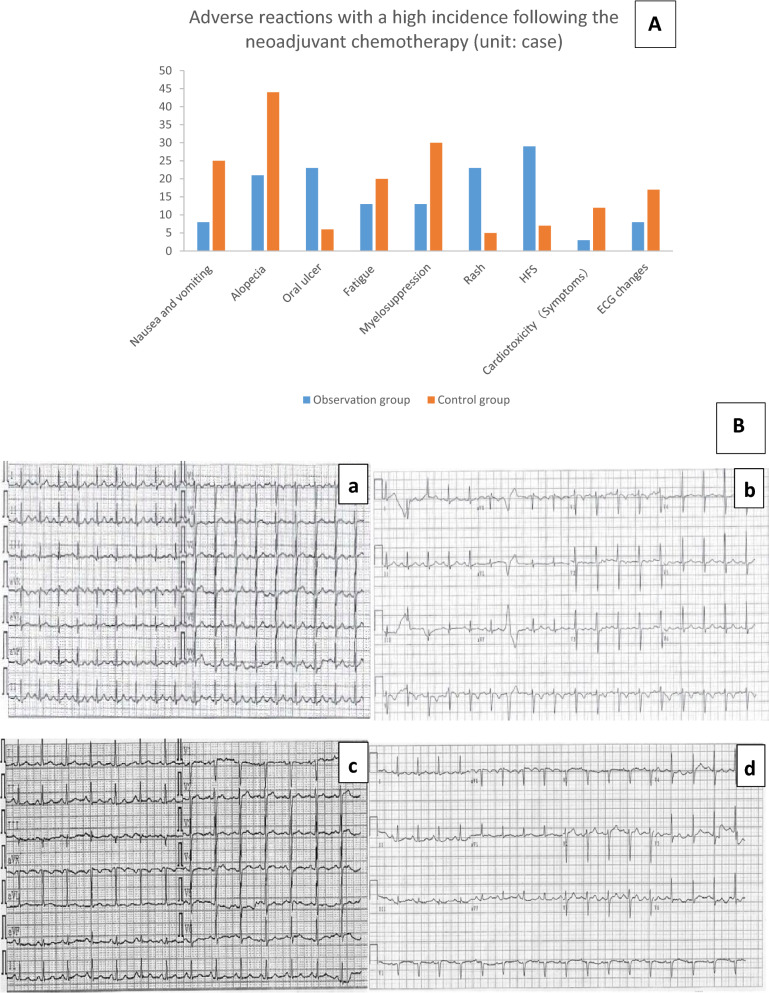
(**A**) The comparison of adverse reactions between observation group and control group; (**B**) ECG comparison. (a) The normal ECG of one patient in the control group before chemotherapy, (b) there was occasional ventricular contraction after chemotherapy; (c,d) were the normal ECG of one patient in the observation group before and after chemotherapy.

The adverse reactions with a high incidence observed following the preoperative neoadjuvant chemotherapy were recorded, mostly in level II or III, as shown in Table 5 and Fig. 2. Compared with the control group, the observation group reported an evidently higher incidence of oral ulcer, rash and hand foot syndrome (HFS), but a significantly lower incidence of nausea, vomiting, alopecia and myelosuppression. Other side effects, except for fatigue which occurred similarly in the two groups, were rare or less severe with a degree of grade I or lower. All of these adverse reactions have been treated by active symptomatic treatment till the full recovery before surgeries.

## Discussion

Over the recent years, new adjuvant treatment has contributed to an increase in the PCR of breast cancer due to the progress of the techniques for comprehensive treatment, which can not only improve the survival rate and prognosis of the patients, but is also associated with the optimization of operation approaches and the scope of resection for patents with breast cancer^2^. In this clinical study, the observation group showed a higher PCR rate and a significant superior efficacy based on Miller-payne grading as compared to the control group. Considering that paclitaxel-albumin, an agent that can effectively improve the PCR rate for patients with triple negative breast cancer according to some studies^15^, was used in both groups in our study, including the four patients who obtained PCR in the observation group, we removed the data of these four patients to eliminate the potential confounding factors for analysis. As a result, the postoperative pathologic differences were still statistically significant between the two groups (*p* = 0.024), indicating a better pathologic response to PLD when compared with traditional anthracycline drugs represented by pirarubicin.

Besides pathologic examinations, clinical assessment also play a particularly important role in evaluating the efficacy of preoperative neoadjuvant chemotherapy. Multiple approaches can be used to measure the changes in tumor size, but some of them are less reliable for a precise assessment, such as clinical palpation, X-ray or common ultrasound examinations^16^. According to the RECIST, CT and MRI can provide the most accurate evaluation on the foci of breast cancer before and after neoadjuvant chemotherapy^17^. However, the high cost has greatly lowered their accessibility in clinical settings where multiple imageological examinations are demanded. Therefore, we used Color Doppler ultrasound which has a high accuracy and acceptable cost in our study as the tool for tumor measurement. The results revealed no evident difference in clinical response between the two groups, suggesting a similar clinical effect of PLD and its control agent.

Cardiac toxicity is considered as one of the major side effects of anthracyclines. To evaluate the cardiotoxicity caused by anthracyclines, LVEF was employed in this study as an indicator. As a result, no significant reduction of LVEF was found in the two groups in comparison with the baseline levels. Nevertheless, monitoring on the clinical symptoms and ECG changes associated with cardiotoxicity revealed a better performance in the observation group, suggesting a superior safety of PLD regarding to cardiotoxicity. As for other adverse reactions, the observation group had a remarkably lower incidence of nausea, vomiting, alopecia, and even myelosuppression which is considered as a life-threatening side effect of anthracyclines. Although a higher incidence of oral ulcer, skin toxicity and HFS was observed in the patients of observation group, they are reversible and controllable. HFS is a characteristic adverse reaction related to PLD^18^, with a typical manifestation of numbness and tingling of hands and feet, which may further progressed into desquamation, scab and ulcer. HFS can be cured by oral administration of medicines or external use of ointment.

Through the comparison of the clinical and pathologic responses as well as the adverse reactions between PLD and the control agents, we can conclude that PLD is a safe and effective chemotherapeutic drug, and thus suggest PLD to be considered as an alternative in developing chemotherapy regimens for breast cancer patients undergoing preoperative neoadjuvant chemotherapy. Different from most of the other studies, our study was based on a relatively large sample size and particularly, 1-to-1 pairing protocol in patient recruiting, which is favorable to a convincing comparison between the observation group and control group due to the high similarity in their demographic and disease characteristics. Besides, comparison between PLD and traditional anthracyclines in curative effect and cardiotoxicity are not common in previous studies in China for breast cancer patients undergoing preoperative neoadjuvant chemotherapy. The limitation of our study was also related to the evaluation on cardiotoxicity, since dexrazosen, a cardioprotective agent according to other study^19^, was used for all the patients treated with anthracyclines to prevent the occurrence of cardiac toxic events, and thus may compromise the results of our study to some extent. Moreover, as the period for observation is not long enough to evaluate the accumulation of the agents, long-term follow-ups are necessary to trace the long-term survival rate and the adverse reactions.
